# A Dual–Mode Nanozyme Assay Based on Photoactivated Mn–Doped Carbon Nanosheets for Rapid Evaluation of Antioxidant Responses in Tea Beverages and Dairy Tea Matrices

**DOI:** 10.3390/foods15142572

**Published:** 2026-07-22

**Authors:** Qiongmeng Lu, Qiuju He, Xiling Fang, Zheng Wei, Qi Zhang, Yaxiong Song, Shijie Li, Shuo Wang

**Affiliations:** 1 School of Grain Science and Technology, Jiangsu University of Science and Technology, Zhenjiang 212100, China; 15961061618@163.com (Q.L.); 19556272487@163.com (Q.H.); fxiling@outlook.com (X.F.); 17603819557@163.com (Z.W.); zhangqi_ibe@163.com (Q.Z.); 2 Tianjin Key Laboratory of Food Science and Health, School of Medicine, Nankai University, Tianjin 300071, China; ysong90@nankai.edu.cn

**Keywords:** Mn–CNSs, dual–mode assay, nanozyme, antioxidant responses, tea beverages, matrix effect

## Abstract

Rapid and matrix–aware evaluation of antioxidant responses in dairy–containing tea beverages remains challenging because milk components may interfere with the detectable activity of tea polyphenols. In this study, a photoactivated Mn–doped carbon nanosheet (Mn–CNS)–based dual–mode nanozyme assay was developed for the rapid evaluation of antioxidant responses in tea beverages and dairy tea matrices. The Mn–CNSs exhibited light–triggered oxidase–like activity toward 3,3′,5,5′–tetramethylbenzidine, enabling synchronized colorimetric and fluorescence readouts within 1 min. Using epigallocatechin gallate as a model antioxidant, the assay showed limits of detection of 0.22 and 0.29 μg/mL in the colorimetric and fluorescence modes, respectively, together with good selectivity and applicability to tea infusions. When applied to milk tea systems, the measurable antioxidant response decreased to 56.9–72.2% of that in corresponding tea infusions, indicating a pronounced matrix–dependent attenuation. Matrix–matched calibration and dual–indicator recovery analysis further distinguished tea infusion recovery from antioxidant response recovery, while molecular docking suggested that β–lactoglobulin–EGCG interactions contributed to the reduced detectable response. These results demonstrate that the proposed dual–mode assay is a rapid tool for antioxidant–response evaluation and highlight the importance of matrix–aware interpretation in complex dairy tea beverages.

## 1. Introduction

Tea beverages are widely consumed because of their sensory appeal and the presence of bioactive compounds, among which tea polyphenols are major contributors to antioxidant responses. Epigallocatechin gallate (EGCG), one of the major catechins in tea, is commonly used as a representative compound for evaluating antioxidant–related responses in tea–based systems [1]. Rapid assessment of antioxidant responses in tea beverages is therefore of considerable interest for beverage analysis, formulation studies, and comparative evaluation of different products. However, in complex beverage matrices, the measurable antioxidant response does not always directly reflect the actual amount of tea–derived polyphenols, particularly when additional components capable of interacting with polyphenols are present. This issue is especially relevant in dairy–containing tea beverages. In milk tea systems, tea polyphenols may interact with milk proteins through noncovalent interactions such as hydrogen bonding, hydrophobic interactions, and van der Waals forces, which may alter the accessibility of phenolic hydroxyl groups and affect their measurable reducing activity [2,3]. Consequently, a conceptual distinction must be drawn between the detectable antioxidant response, the fraction of polyphenols that remain reactive and accessible under assay conditions, and the actual polyphenol content, which represents the total inventory introduced during formulation. This distinction is methodologically significant: while conventional assays may report the former, quality assessment and nutritional labeling often implicitly assume the latter. Therefore, an analytical approach that can not only rapidly quantify antioxidant responses but also explicitly address the divergence between detectable signal and actual content in matrix–dependent contexts would provide a more robust basis for interpreting polyphenol–related functionality in real beverage systems.

Conventional analytical methods, such as high–performance liquid chromatography (HPLC) and Folin–Ciocalteu assays, remain important for laboratory–based analysis of tea catechins and total phenolics, but they are less suited for rapid screening because of their instrumentation requirements, labor–intensive procedures, and limited practicality for routine analysis [4,5].

In recent years, nanozymes have attracted increasing attention in analytical chemistry because of their enzyme–mimetic catalytic activity, good stability, tunable catalytic performance, and low preparation cost [6]. Compared with natural enzymes, nanozymes are generally more tolerant to environmental variations and are therefore suitable for rapid analysis in complex food matrices. Among them, photoactivated nanozymes are particularly attractive because their catalytic activity can be triggered on demand by light irradiation [7,8]. This light–controlled catalytic process provides temporal regulation of the reaction and is beneficial for rapid color development in antioxidant–response assays.

Carbon–based nanomaterials are useful platforms for constructing multifunctional sensing systems because of their tunable surface chemistry, favorable optical properties, and structural adaptability [9,10]. In particular, carbon nanosheets provide a large accessible surface and abundant surface sites, which can facilitate substrate contact and interfacial electron transfer. The introduction of Mn species can further regulate the electronic structure of carbon nanosheets and provide redox–active Mn sites, thereby enhancing oxidase–like catalytic behavior [11,12]. Meanwhile, the intrinsic fluorescence of carbon–based materials enables the construction of dual–mode sensing platforms that combine catalytic colorimetric responses with fluorescence readouts [13,14]. Therefore, Mn–doped carbon nanosheets are suitable for the present assay because they integrate photoactivated oxidase–like activity, fluorescence signal output, and surface–accessible catalytic sites, which may improve the reliability of antioxidant response analysis in complex tea and dairy tea matrices [15,16].

**Scheme 1:**
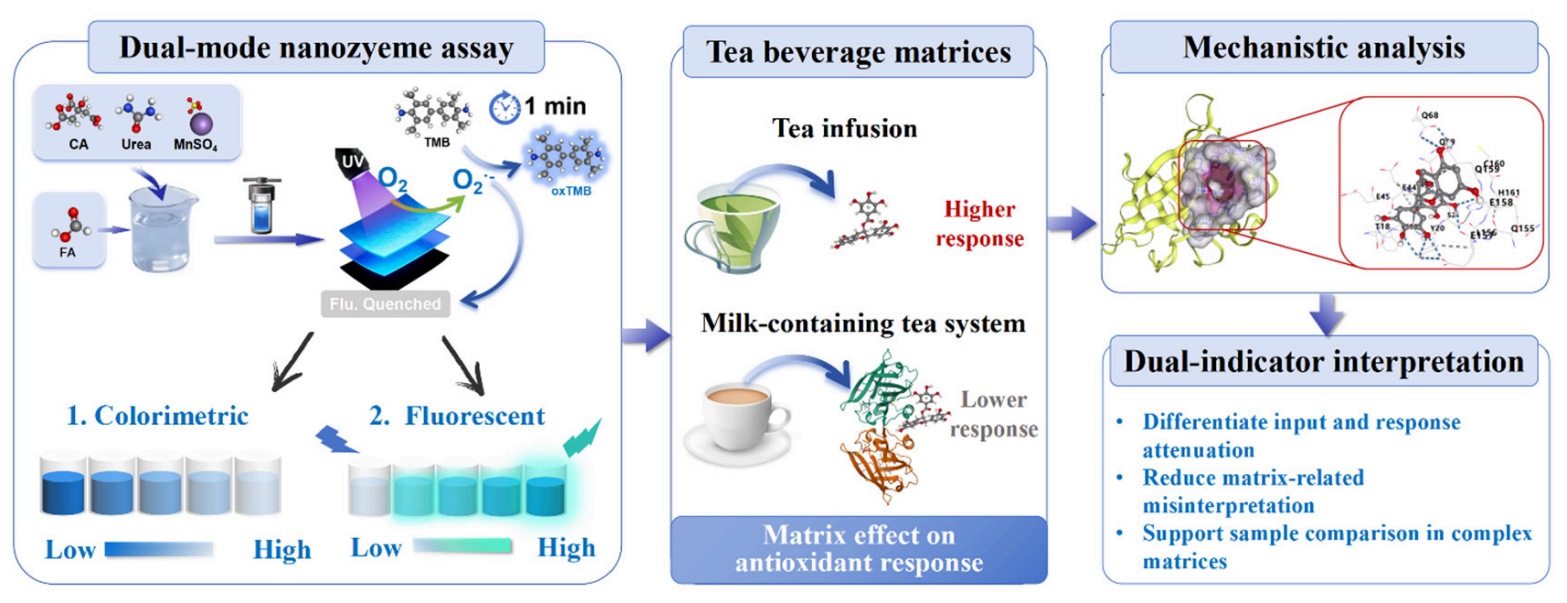
Schematic illustration of a matrix–aware dual–mode nanozyme assay for antioxidant response analysis in tea beverages and dairy tea matrices.

In this study, we developed a photoactivated Mn–CNSs–based dual–mode nanozyme assay for rapid evaluation of antioxidant–related responses in tea beverages (Scheme 1). The Mn–CNSs exhibited light–triggered oxidase–like activity toward TMB and enabled synchronized colorimetric and fluorescence readouts within 1 min. Using EGCG as a model antioxidant, a rapid dual–mode colorimetric/fluorescence assay was established for the evaluation of antioxidant response in tea beverages. The method was first validated in simple tea infusions and then extended to dairy–containing tea systems to test the hypothesis that protein–polyphenol interactions cause a measurable divergence between detectable antioxidant response and actual polyphenol content. Molecular docking with β–lactoglobulin was performed to provide mechanistic support for this suppression. Furthermore, a matrix–matched calibration strategy coupled with dual–indicator recovery analysis was implemented to disentangle tea infusion recovery from antioxidant response recovery, thereby enabling matrix–aware interpretation of polyphenol functionality in complex beverage systems. This work provides not only a convenient analytical tool for antioxidant response analysis in tea beverages, but also a practical framework for distinguishing recoverable input from functionally accessible output in polyphenol–protein interaction systems.

## 2. Materials and Methods

### 2.1. Materials

Manganese sulfate (MnSO_4_), urea, citric acid, dimethyl sulfoxide (DMSO), N,N–dimethylformamide (DMF), and absolute ethanol were purchased from Sinopharm Chemical Reagent Co., Ltd. (Shanghai, China). TMB, 2–(N–morpholino)ethanesulfonic acid monohydrate (MES), epigallocatechin gallate (EGCG), Gallocatechin gallate (GCG), catechin (EC), Lactoferrin (LF), β–lactoglobulin (β–LG), casein (CN), ascorbic acid (VC), formic acid, p–benzoquinone (PBQ), tert–butanol (TBA), L–histidine (L–His), hydrogen peroxide (H_2_O_2_), and ferrous sulfate (FeSO_4_) were obtained from Shanghai Macklin Biochemical Co., Ltd. (Shanghai, China). The UV lamp (365 nm, 2 W/cm^2^) was purchased from Zhongshan Miaogu Lighting Co., Ltd. (Zhongshan, China). Green tea, jasmine tea and black tea were purchased from Lipton (Unilever (China) Investment Co., Ltd., Shanghai, China). Pu’er tea were bought from local supermarket, and milk was purchased from Mengniu Dairy (China Mengniu Dairy Co., Ltd., Hohhot, Inner Mongolia, China; raw cow milk as the only ingredient; protein, 3.2 g/100 mL; fat, 4.0 g/100 mL; no declared additives or stabilizers).

### 2.2. Synthesis of Mn–CNSs

Mn–CNSs were synthesized via a modified solvothermal method, with reference to reported solvothermal preparation of Mn–doped carbon dots [17]. Briefly, 1 g of citric acid monohydrate, 1 g of manganese sulfate monohydrate, and 2 g of urea were dissolved in 10 mL of formic acid by ultrasonication for 1 h. The mixture was transferred to a Teflon–lined autoclave and heated at 180 °C for 6 h. After cooling to room temperature, the supernatant was discarded, and the precipitate was washed with ethanol three times. The product was collected by centrifugation at 10,000 rpm for 10 min and redispersed in MES buffer to obtain a uniformly dispersed solution.

### 2.3. Analysis of Oxidase–like Activity of Mn–CNSs

TMB was used as the chromogenic substrate to evaluate the oxidase–like activity of Mn–CNSs. In a typical assay, 60 μL of Mn–CNSs solution (diluted with 50 mM MES buffer) was mixed with 50 μL of 2.5 mM TMB solution in a 96–well plate. The reaction was performed in the MES buffer system. After irradiation for 60 s, the oxidation of TMB was monitored by the blue color appearance, and the absorbance of the oxidized product (oxTMB) was recorded at 652 nm using a UV–Vis spectrophotometer (Implen GmbH, Munich, Germany).

### 2.4. Determination of Catalytic Kinetic Parameters of Nanozymes for TMB

In a 96–well plate, 50 μL of TMB solutions with different concentrations (0.25, 0.5, 0.75, 1, 1.25, 1.5, 1.75, 2, 2.25, and 2.5 mM) were added separately, followed by 60 μL of prepared Mn–CNSs solution (0.2 mg/mL). Irradiation was performed at a distance of 5 cm from the light source at room temperature for a total of 120 s. After each irradiation, the plate was quickly transferred to a microplate reader to measure the UV–Vis absorbance at 652 nm. The initial reaction rate (v) was obtained from the linear slope of the time–dependent absorbance curves at 652 nm under different TMB concentrations. The kinetic parameters were determined using the Michaelis–Menten equation, v = Vmax[S]/(Km + [S]), and the Lineweaver–Burk equation, 1/v = Km/(Vmax[S]) + 1/Vmax, where v is the initial reaction rate, Vmax is the maximum reaction rate, Km is the Michaelis constant, and [S] is the TMB concentration.

### 2.5. Detection of Antioxidant Responses of EGCG

EGCG standard solutions with concentrations of 0.01, 0.004, 0.002, 0.001, 0.0004 and 0.0002 mg/mL (100 μL each) were used as controls, and samples were diluted before detection. For both colorimetric and fluorometric detection, 60 μL of Mn–CNSs solution and 50 μL of 2.5 mM TMB solution were first mixed in the MES buffer system. After color development under light irradiation for 1 min, 100 μL of EGCG standard solution or sample solution was added to the reaction system. The colorimetric signal was recorded at 652 nm, while the fluorescence signal was collected at an excitation wavelength of 380 nm and an emission wavelength of 450 nm. The colorimetric response (ΔA) and fluorescence recovery response (ΔF) were calculated as ΔA = A_0_ − A and ΔF = F − F_0_, respectively, where A_0_/F_0_ and A/F represent the absorbance at 652 nm or fluorescence intensity at 450 nm of the blank system and the system containing EGCG or sample, respectively. The LOD was calculated as 3σ/k, where σ is the standard deviation of the blank signal and k is the slope of the calibration curve.

### 2.6. Analysis of Real Samples

For tea sample preparation, 2.0 g of tea fragments were soaked in 50 mL of room–temperature distilled water for 5 min. After soaking, the tea leaves were removed, and the obtained tea infusion was collected for subsequent analysis. For milk tea samples, the tea infusion and milk were mixed at the preset volume ratios and allowed to stand before detection. The prepared tea infusion or milk tea sample was introduced into the detection system according to the procedure described in Section 2.5, and the antioxidant response was evaluated using both colorimetric and fluorometric signals. All samples were processed in duplicate and subsequently analyzed in triplicate.

### 2.7. Molecular Docking Between β–LG and EGCG

The blind docking experiment of protein–ligand was performed via the CB–DOCK2 platform (https://cadd.labshare.cn/cb-dock2/php/index.php, accessed: 13 April 2026). The 3D structure of β–LG was retrieved from the Protein Data Bank (PDB ID: 1B0O), and the PubChem Compound Identifier (CID) of the EGCG is 65064. The experimental procedure followed the standard protocol of CB–DOCK2, and the identification and localization of binding cavities were completed by the curvature–based detection algorithm built into the platform before blind docking.

### 2.8. Statistical Analysis

All measurements were performed in triplicate, and the results are expressed as mean ± standard deviation (SD). Data fitting and statistical analysis were performed using Origin. For comparisons among multiple groups, statistical significance was evaluated by one–way analysis of variance (ANOVA) followed by Tukey’s multiple–comparison test, and *p* < 0.05 was considered statistically significant.

## 3. Results and Discussion

### 3.1. Structural Characterization of Mn–CNSs

**Figure 1:**
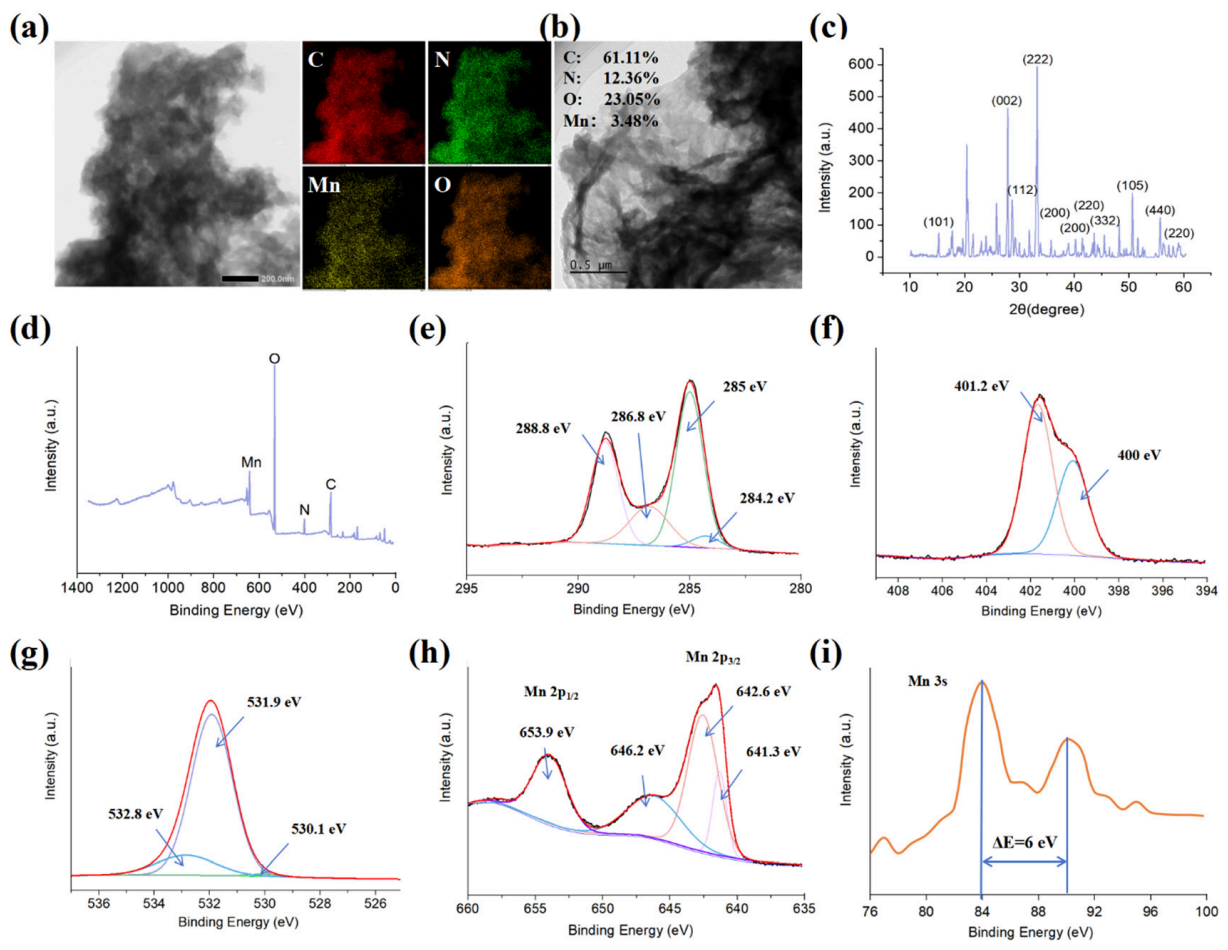
Structural and compositional characterization of Mn–CNSs. (**a**) TEM Mapping image (scale bar: 200 nm); (**b**) TEM image (scale bar: 500 nm) and elemental composition analysis; (**c**) XRD spectrum; (**d**) Full–scan XPS spectrum; (**e**–**i**) High–resolution XPS spectra: (**e**) C 1s; (**f**) N 1s; (**g**) O 1s; (**h**) Mn 2p and (**i**) Mn 3s.

Structural and compositional characterization is essential for understanding the origin of the photoactivated oxidase–like activity of Mn–CNSs and for confirming the successful formation of the sensing nanomaterial. The catalytic performance of Mn–CNSs is expected to be related to their nanosheet morphology, elemental distribution, crystalline composition, surface chemical states, Mn valence states, and oxygen–related surface species. Therefore, TEM and EDS were used to evaluate the morphology and elemental distribution, XRD was employed to identify the crystalline phases, and XPS was conducted to analyze the surface bonding states and Mn oxidation states. Transmission electron microscopy (TEM) images (Figure 1a,b) and elemental distribution analysis of the synthesized nanomaterial showed that the material exhibits a fluffy nano–sized agglomerated structure with porous or thin–layer assembly characteristics overall. This structure is conducive to increasing the specific surface area and providing sufficient active sites for catalytic reactions. The elemental mapping images intuitively demonstrate the uniform distribution of C (red), N (green), Mn (yellow), and O (orange) in the material, confirming the uniform nanoscale composite of g–C_3_N_4_–like components and manganese–based oxides, which lays a structural foundation for their synergistic effect in photoactivated oxidase catalysis. The TEM images on the right show that Mn–CNSs present layered/sheet–like structural features, consistent with the typical layered morphology of g–C_3_N_4_–like materials. Energy–dispersive spectroscopy (EDS) analysis revealed the elemental contents: C 61.11%, N 12.36%, O 23.05%, and Mn 3.48%. The high proportions of C and N are consistent with the elemental composition of g–C_3_N_4_–like materials, the presence of Mn corresponds to manganese–based oxide components, and O originates from both manganese–based oxides and oxygen–containing functional groups on the material surface (e.g., adsorbed oxygen near oxygen vacancies).

X–ray diffraction (XRD) analysis results (Figure 1c) showed that the characteristic diffraction peaks of the sample can be assigned to specific crystal planes of different substances: the peaks at 15.2°, 28.6°, and 50.7° correspond to the (101), (112), and (105) crystal planes of tetragonal spinel Mn_3_O_4_ (JCPDS 24–0734) [18]; the sharp peaks at 23.2°, 32.9°, 38.2°, 45.3°, 49.3°, 55.1°, and 65.7° can be accurately indexed to the (211), (222), (400), (332), (431), (440), and (622) crystal planes of α–Mn_2_O_3_ (JCPDS 41–1442) [19,20]; the sharp peak at 27.7° corresponds to the (002) crystal plane of graphitized–C (JCPDS 87–1526), and the sharp and intense (002) peak indicates a high degree of graphitization of the carbon–nitrogen layers [21]; the sharp peaks at 35.08°, 40.98°, and 59.06° are completely matched with the (111), (200), and (220) crystal planes of rock–salt phase MnO (JCPDS 07–0230) [22,23], respectively. The formation of low–valent manganese oxides provides a basis for the electron transfer of Mn–CNSs and the enhancement of their oxidase–like activity. These results suggest the apparent multiphase nature of Mn–CNSs, in which carbon–based domains coexist with MnO, Mn_3_O_4_, and α–Mn_2_O_3_ phases.

X–ray photoelectron spectroscopy (XPS) analysis results (Figure 1d–i) showed that the XPS data of C element revealed peaks at 284.27, 285.00, 286.82, and 288.80 eV can be assigned to C–C sp^2^, C–C sp^3^, C–O, and C=O species, respectively, with corresponding FWHM values of 1.431, 1.431, 2.069, and 1.431 eV and relative areas of 3.63%, 47.50%, 17.22%, and 31.21%; a weak component at 293.50 eV, accounting for only 0.44%, was attributed to a shake–up/satellite contribution (Figure 1e) [21]. The N 1s spectrum was fitted into two components at 400.09 and 401.68 eV, corresponding to C–N–C/pyrrolic N and graphitic or coordinated N species, with FWHM values of 1.633 and 1.575 eV and relative areas of 39.55% and 60.45%, respectively (Figure 1f) [24]. In the O 1s spectrum (Figure 1g), the main fitted peaks at 530.05, 531.92, and 532.85 eV were assigned to Mn–O, oxygen vacancies/defective oxygen, and adsorbed or surface oxygen species, respectively, with FWHM values of 1.249, 1.743, and 2.479 eV and relative areas of 0.82%, 83.15%, and 14.53% [25]. The Mn 2p spectrum showed fitted components at 641.32 and 642.57 eV, corresponding to Mn^2+^ and Mn^3+^ species in the Mn 2p_3_/_2_ region, with FWHM values of 1.202 and 2.635 eV and relative areas of 11.67% and 41.74%, respectively [21,26]. In addition, the peak at 646.18 eV was assigned to the satellite feature of MnO, while the component at 653.97 eV corresponded to Mn 2p_1_/_2_ [27] (Figure 1h). Furthermore, the XPS 3s spectrum exhibits a double–peak splitting feature due to electron exchange between the 3s and 3d orbitals, and the exchange interaction energy reflected by the peak splitting width can be used to roughly determine the material phase. The measured splitting width of 6 eV in this study is consistent with the reported splitting width range of MnO [26] (Figure 1i). These fitted results indicate the coexistence of Mn^2+^/Mn^3+^ species, abundant oxygen–related surface defects, and carbon/nitrogen–containing surface groups, further supporting the multiphase nature of Mn–CNSs and their potential role in promoting interfacial electron transfer during photoactivated oxidase–like catalysis.

### 3.2. Optical and Catalytic Characterization of Mn–CNSs

**Figure 2:**
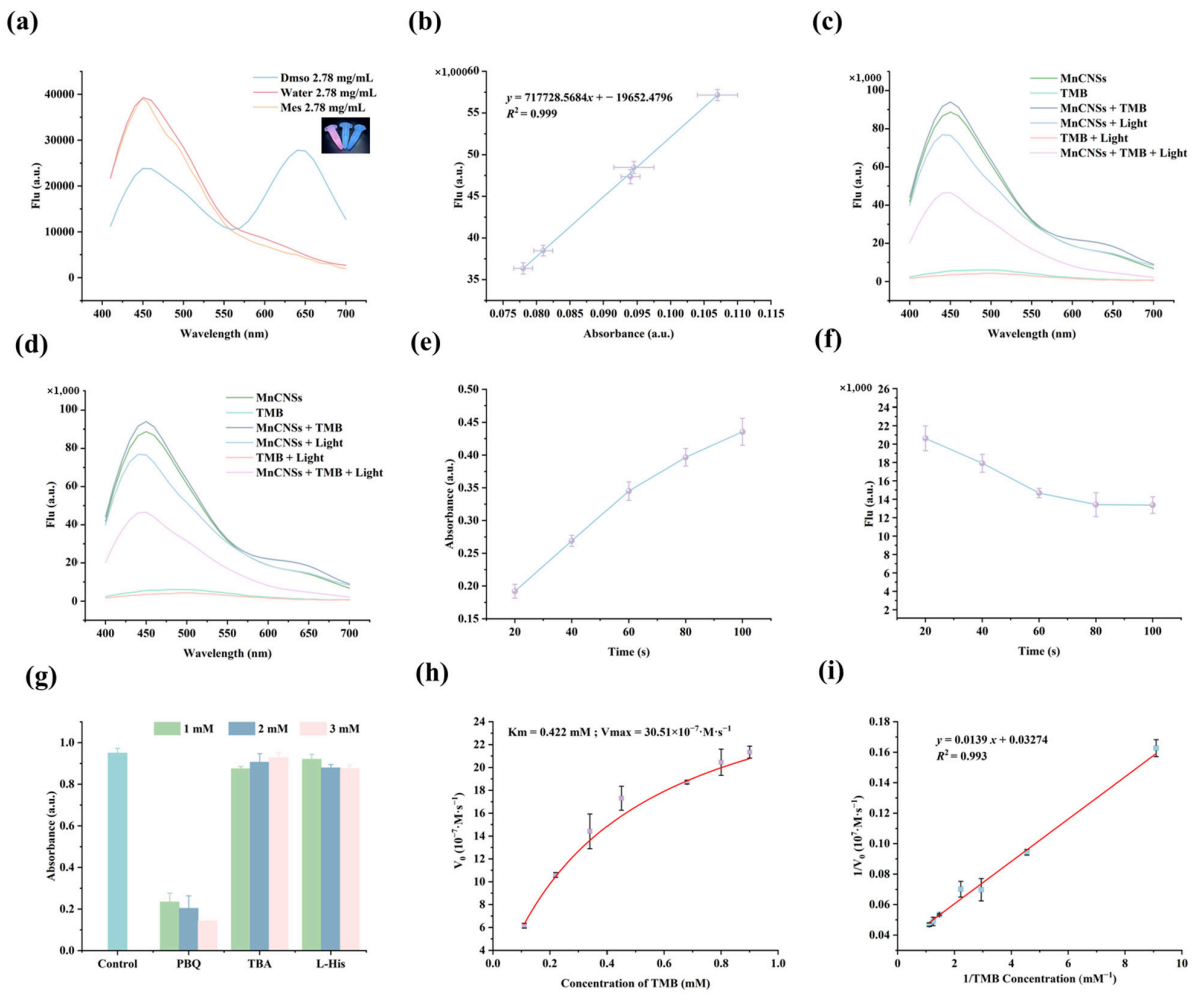
Optical properties, photoactivated oxidase–like activity, and kinetic characterization of Mn–CNSs. (**a**) Fluorescence emission spectra of Mn–CNSs in dimethyl sulfoxide (DMSO), water, and MES buffer (inset: fluorescence photographs of Mn–CNSs dispersed in DMSO, water, and MES buffer, respectively); (**b**) Correlation curve between absorbance and fluorescence intensity of Mn–CNSs in the MES buffer system; (**c**) UV–Vis absorption and (**d**) fluorescence spectra of photoactivated nanozymes reaction systems; (**e**) Time–dependent absorbance and (**f**) fluorescence changes of the nanozyme–catalyzed reaction systems; (**g**) Effect of ROS scavengers; (**h**) Michaelis–Menten kinetic curve and (**i**) Lineweaver–Burk double reciprocal plot of Mn–CNSs catalyzing TMB oxidation.

Optical and catalytic characterizations are crucial for establishing the Mn–CNSs–based dual–mode assay. Therefore, UV–Vis and fluorescence spectroscopy were employed to evaluate the absorption, emission, and signal response behaviors of Mn–CNSs. Additionally, reactive oxygen species (ROS) scavenging assays were conducted to identify the primary ROS involved in the catalytic process, and kinetic analyses were performed to quantify the catalytic affinity and efficiency toward TMB (Figure 2). As shown by the absorption spectra (Figure S1), Mn–CNSs exhibited the highest overall absorbance in DMSO, whereas the absorbance in water and MES buffer was relatively weaker. However, all three systems displayed a broad absorption band centered at approximately 550–580 nm. These results indicate that the fundamental electronic transition characteristics of Mn–CNSs were retained in different media, while the absorption intensity and long–wavelength absorption behavior were markedly affected by solvent polarity. The fluorescence spectra further revealed the regulatory effect of different solvents on the emissive centers of Mn–CNSs (Figure 2a) [28]. In water and MES buffer, Mn–CNSs exhibited a dominant blue emission peak centered at approximately 450 nm, while the long–wavelength emission was relatively weak. In contrast, in DMSO the emission around 450 nm was significantly reduced, whereas a pronounced long–wavelength emission band appeared at approximately 630–650 nm. These results suggest that Mn–CNSs contain at least two distinct emissive centers. The short–wavelength blue emission is more likely associated with π–π* transitions of the carbon framework or surface–related states, whereas the long–wavelength emission may originate from defect states or metal–related energy levels introduced by Mn doping [29,30]. As a strongly polar aprotic solvent, DMSO may better stabilize the long–wavelength emissive centers, thereby enhancing their emission intensity [31]. In aqueous systems, by contrast, hydrogen–bonding interactions and surface solvation effects make the blue emission dominant.

To further evaluate the optical response of Mn–CNSs under working conditions, the relationship between absorbance and fluorescence intensity in MES buffer was examined (Figure 2b and Figure S2). In DMSO (Figure S2a) and MES (Figure 2b) buffer systems, the fluorescence intensity shows a good linear correlation with absorbance, with correlation coefficients (R^2^) of the fitting equations being 0.997 and 0.999, respectively. This indicates that Mn–CNSs exhibit excellent uniformity and dilution compliance in both organic solvents and buffer systems. In the aqueous system (Figure S2b), the change in fluorescence intensity with absorbance presents a logarithmic relationship (R^2^ = 0.999), which further verifies that water regulates the surface state of Mn–CNSs through hydrogen bonding, causing the fluorescence response to deviate from the simple linear increase law while still maintaining a high correlation. Therefore, subsequent experiments were conducted in MES buffer.

In addition, the optical properties of Mn–CNSs under different synthesis conditions were also characterized. UV–Vis spectroscopy showed that Mn–CNSs exhibit a broad absorption feature in the range of 300–900 nm (Figure S3a). The characteristic differences in the intensity and position of absorption and fluorescence peaks indicate that the synthesis temperature and time have a significant regulatory effect on the optical response of the material. The UV absorption peak redshifts from 460 nm to 562 nm with increasing temperature, and the absorbance value increases with prolonged time. This is attributed to the gradual transformation of amorphous or low–graphitized carbon–based structure into more ordered sp^2^–hybridized carbon layer–stacked structures at high temperatures, which essentially reduces the electron transition energy barrier. Fluorescence spectroscopy in aqueous solution further revealed performance differences. As the temperature increases, the solution fluorescence transitions from orange–yellow to green and finally to blue (inset of Figure S3b). The long–wavelength fluorescence at 600–700 nm is usually directly related to the electron transition of low–valent manganese (Mn^2+^). Fluorescence spectra show that when the temperature rises to 200 °C, the fluorescence at 600–700 nm completely disappears, which may be due to the oxidation of Mn^2+^ to high–valent states, the disappearance of d–d transitions, and excessive graphitization. In contrast, the blue fluorescence at 450 nm mainly originates from the π→π* transition of the carbon–based conjugated structure. With increasing temperature and prolonged time, the peak position at 450 nm shows no significant shift, indicating that the nature of the electron transition remains unchanged. Notably, the condition of heating at 180 °C for 6 h achieves the highest peak intensity at 450 nm while retaining the fluorescence signal at 600–700 nm. This indicates that under this condition, not only the formation of ordered sp^2^ conjugated structures in the carbon matrix is promoted, but also manganese atoms are uniformly doped to form stable coordination sites, with the excitation spectrum peak located at 380 nm.

The storage stability of Mn–CNSs was further evaluated by investigating the effects of pH and storage light conditions on their optical properties. Under pH 3–9 (Figure S4), the absorption of the sample in the visible region remains generally stable, with only a slight decrease at the highly alkaline end. In the fluorescence mode excited at 380 nm, the emission peak position is consistent, while the intensity varies with pH, reaching the maximum under neutral conditions (pH 7). A further comparison between samples stored under light and dark conditions (Figure S5) shows that their absorption curves at 300–900 nm are basically overlapping, and the emission peak at 450 nm excited at 380 nm also shows no shift, with only a slightly higher intensity in the light–exposed group. The above results indicate that Mn–CNSs have good optical stability under conventional storage environments, and neutral pH can achieve the optimal signal–to–noise ratio and response, making them suitable as working conditions for the detection of actual foods and beverages.

To systematically evaluate the photoactivated oxidase–mimicking activity of Mn–CNSs, comprehensive characterization was conducted through spectroscopic analysis, mechanism investigation, and kinetic studies. UV–Vis absorption spectroscopy (Figure 2c) revealed that only when Mn–CNSs and the substrate TMB coexisted under light irradiation did the system exhibit a characteristic absorption peak at 652 nm corresponding to oxTMB, confirming its light–triggered catalytic oxidation capability. The catalytic activity may be associated with the unique material structure and surface active sites of Mn–CNSs. Upon photoexcitation, the valence–state variation in surface Mn species (Mn^2+^/Mn^3+^) may facilitate interfacial electron transfer and promote the activation of dissolved O_2_, which could contribute to ROS–mediated TMB oxidation. In addition, the layered conjugated structure may favor the separation of photogenerated electron–hole pairs, while C–N bonds could facilitate charge migration, thereby improving the photoactivated oxidase–like activity.

Fluorescence spectroscopy (Figure 2d) showed that this catalytic process was accompanied by significant quenching of the intrinsic fluorescence of Mn–CNSs (emission peak at 450 nm). Notably, the maximum absorption peak of oxTMB does not overlap with the maximum emission peak of Mn–CNSs, and oxTMB exhibits no detectable fluorescence in the 400–720 nm range. After light irradiation, the emission intensity of the system was overall lower than that of pure Mn–CNSs, while the shape of the emission band remained consistent (Figure S6). Therefore, the fluorescence quenching of Mn–CNSs is not caused by an inner filter effect. Investigation of the fluorescence quenching mechanism via introduction of the Fenton reaction (Figure S7) demonstrated that the Fenton reaction significantly quenched the fluorescence of Mn–CNSs, whereas in the presence of EGCG (which scavenges ROS), the fluorescence of Mn–CNSs showed no obvious attenuation. This confirms that the fluorescence of Mn–CNSs is ROS–dependent. The influence of the synthesis process on enzyme activity was also verified (Figure S8a,b). The results showed that Mn–CNSs prepared under the conditions of 180 °C for 6 h exhibited the best catalytic performance, with the strongest absorption intensity at 652 nm and the most significant fluorescence quenching effect. This is consistent with the optical property characterization results, indicating that moderate carbonization and surface state modulation can significantly enhance the photoactivated nanozyme catalytic activity of Mn–CNSs.

When examining the effect of light irradiation time on the reaction (Figure 2e,f), it was found that as the irradiation time increased from 20 s to 100 s, the absorbance of the system gradually increased while the fluorescence intensity correspondingly decreased, displaying typical time–dependent catalytic characteristics. When irradiation exceeded 60 s, the signal essentially stabilized, indicating that the reaction approached equilibrium. Further scavenger experiments (Figure 2g) suggested that ·O_2_^–^ may play a dominant role in TMB oxidation, whereas ·OH and ^1^O_2_ appeared to make relatively minor contributions. Kinetic studies (Figure 2h,i) revealed that the catalytic reaction follows the Michaelis–Menten model. The Michaelis constant (Km) was determined to be 0.422 mM, and the maximum reaction rate (Vmax) was 30.51 × 10^–7^ M·s^–1^, demonstrating that Mn–CNSs possess high affinity and catalytic efficiency for TMB, outperforming most reported oxidase–mimicking nanomaterials. Through condition optimization (Figure S9), the optimal amounts of Mn–CNSs and TMB were determined to be 60 μL and 50 μL, respectively. Compared to the reported nanozymes with oxidase–mimicking activity (Table 1), the photoactivated nanocatalyst Mn–CNSs exhibits stronger substrate affinity and higher catalytic efficiency, achieving complete substrate conversion within 1 min. In summary, as an efficient photoactivated nanozyme, Mn–CNSs demonstrates promising potential for rapid and highly sensitive bioanalytical applications.

### 3.3. Analytical Performance for EGCG–Equivalent Antioxidant Response

**Figure 3:**
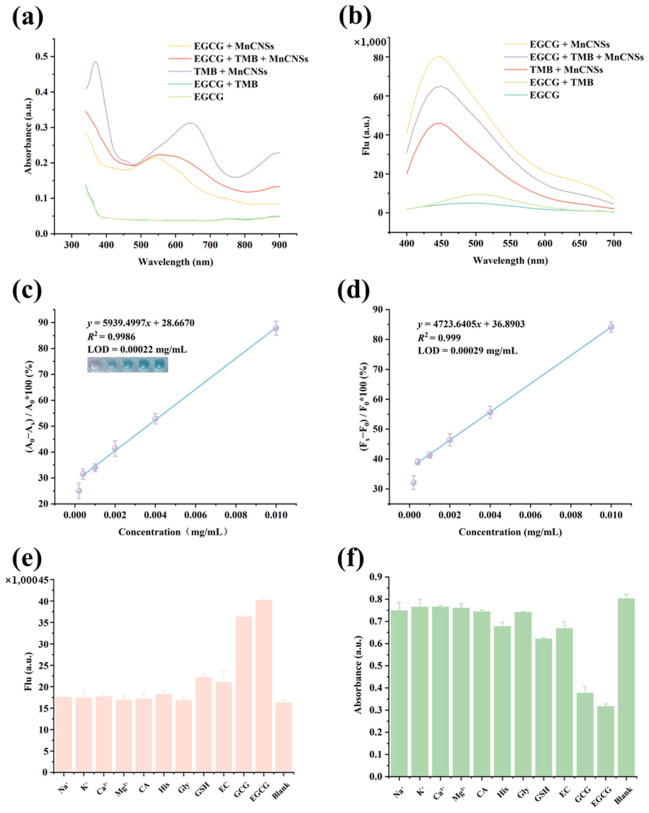
Construction and analytical performance of the Mn–CNSs–based dual–mode assay for EGCG–equivalent antioxidant response. (**a**) UV–Vis absorption and (**b**) fluorescence emission spectra for feasibility verification of antioxidant response evaluation using EGCG as a model antioxidant; (**c**) calibration curve in colorimetric mode; (**d**) calibration curve in fluorescence mode; (**e**) selectivity analysis in colorimetric mode; (**f**) selectivity analysis in fluorescence mode.

Under optimized conditions, we systematically evaluated the detection performance of the Mn–CNSs–based photoactivated oxidase system for antioxidants (calculated as EGCG equivalents). First, the colorimetric response of the system was assessed via UV–Vis absorption spectroscopy (Figure 3a). After the addition of EGCG, the absorption peak intensity of oxTMB decreased significantly, and the blue color of the solution weakened. In the fluorescence mode (Figure 3b), Mn–CNSs exhibited stable intrinsic fluorescence at 450 nm when excited at 380 nm. Upon the addition of TMB and light irradiation, the fluorescence intensity decreased markedly. The introduction of EGCG, however, restored the fluorescence signal almost to its initial level. This trend of fluorescence recovery aligned well with the decrease in absorption signal, confirming that EGCG can synchronously regulate both light absorption and fluorescence emission signals by modulating the redox state of the system, thereby establishing an effective dual–mode response.

In the colorimetric mode (Figure 3c), the EGCG concentration showed a linear negative correlation with the absorbance at 652 nm, with a correlation coefficient R^2^ = 0.9997 and a limit of detection (LOD, A0–3σ) of 0.00022 mg/mL (0.22 μg/mL). As illustrated in the inset, the solution color gradually faded from dark blue with increasing EGCG concentration, consistent with the spectral trend. In the fluorescence mode (Figure 3d), the emission intensity of the system gradually increased with rising EGCG concentration, yielding an LOD (F0 + 3σ) of 0.00029 mg/mL (0.29 μg/mL).

### 3.4. Direct Determination of Antioxidant–Related Response in Tea Infusions

Prior to direct quantification, the background absorption of different beverage matrices at the analytical wavelength (652 nm) was evaluated (Figure S10a). The diluted green tea infusion exhibited negligible absorption at 652 nm, indicating minimal matrix interference with the oxTMB signal. Therefore, the tea infusion was analyzed by simple dilution. The antioxidant response (expressed as EGCG equivalents) in self–prepared Green tea, Jasmine tea, Black tea, and Pu’er tea were determined as 814.3, 652.0, 421.7, and 263.6 μg/mL, respectively. The EGCG–equivalent concentrations obtained by the proposed method were slightly higher than those measured using the commercial total phenol assay kit (by approximately 7.67–15.48%). A possible explanation is that, besides phenolic compounds, tea infusions also contain small amounts of other reducing substances, such as reducing sugars, which may contribute to the analytical response. Nevertheless, the overall agreement between the two methods supports the use of EGCG–equivalent values as a reasonable indicator for evaluating antioxidant–related levels in tea infusions (Figure S10b).

**Figure 4:**
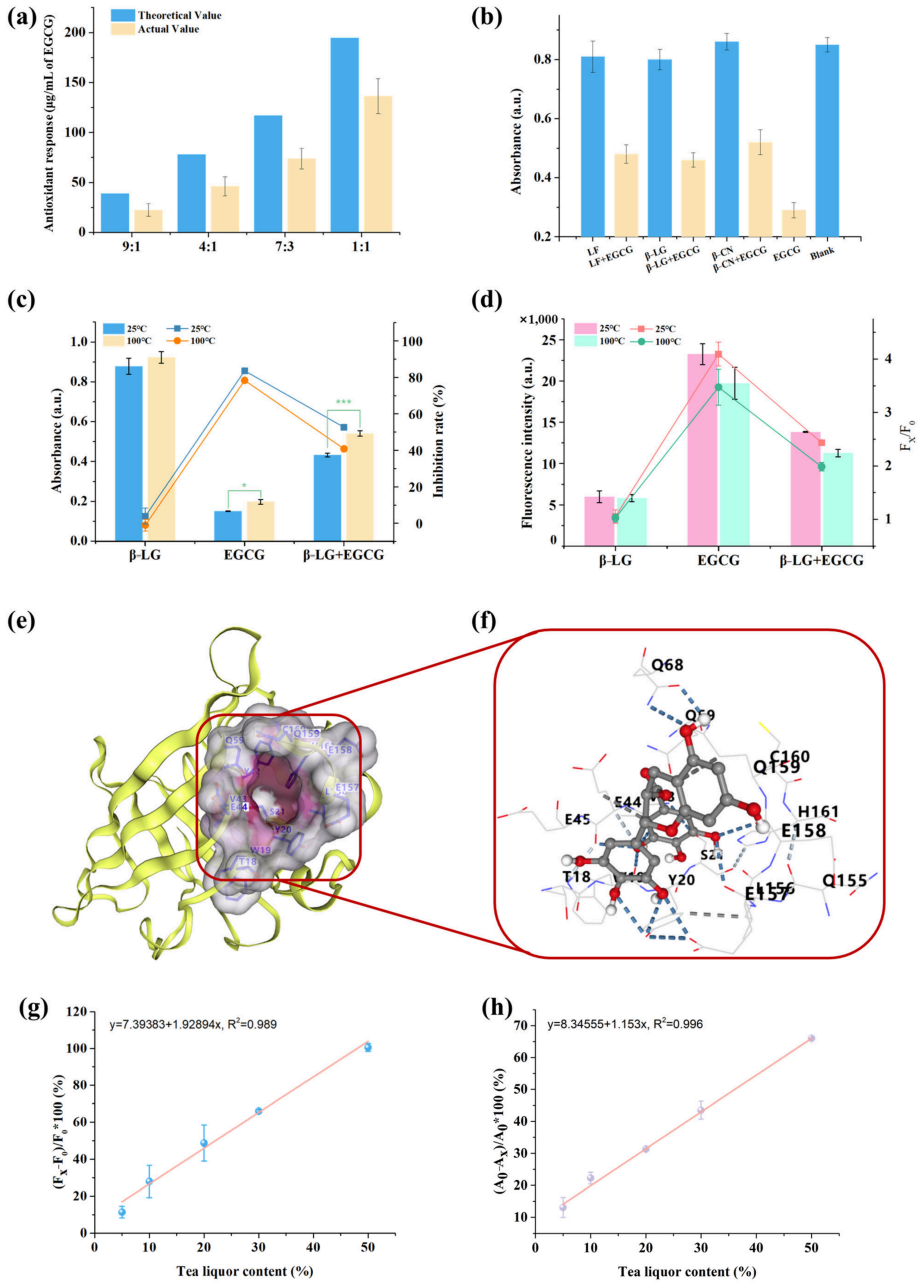
Matrix–aware interpretation of antioxidant responses in dairy tea systems based on model analysis, β–LG–EGCG interaction, and matrix–matched calibration. (**a**) Comparison of theoretical and measured antioxidant responses in milk tea samples with different milk–to–tea ratios; (**b**) Effect of milk proteins on the measurable antioxidant response of EGCG; (**c**) Effect of temperature on the measurable antioxidant response of β–LG, EGCG, and β–LG + EGCG systems (colorimetric mode), the value bars with nsare not significant, asterisk denotes statistically significant differences * *p* < 0.05; *** *p* < 0.001; (**d**) Evaluation of temperature effects on the antioxidant capacity of β–LG, EGCG, and β–LG + EGCG systems (fluorescence mode); (**e**) Active cavity of β–LG for EGCG binding; (**f**) Interaction details of the β–LG–EGCG complex; (**g**) Matrix–matched calibration curve for tea infusion addition in milk (colorimetric mode); (**h**) Matrix–matched calibration curve (fluorescence mode).

In contrast, both milk and milk tea exhibited pronounced background absorption at 652 nm (Figure S10a), which precluded direct colorimetric quantification without proper blank correction. However, even after background subtraction, the measurable antioxidant response in milk tea was substantially lower (only 56.9–72.2%) of that observed in the corresponding tea infusion. This marked suppression cannot be attributed to analytical interference from background absorption (which would typically inflate, not diminish, the apparent signal); rather, it reflects a genuine matrix–dependent attenuation effect. The central issue is therefore not merely elevated background absorption, but that specific binding between milk proteins and tea polyphenols sequesters the latter from participating in the chromogenic reaction. Model–system experiments further support this interpretation (Figure 4b) that EGCG showed markedly reduced detectable activity in the presence of milk proteins, confirming that the attenuation in milk tea arises from specific protein–polyphenol interactions rather than from dilution alone.

β–lactoglobulin (β–LG) was selected as a representative for in–depth analysis (Figure 4c,d). At 25 °C, β–LG inhibited the antioxidant response of EGCG by 37%, and the inhibition increased to 47.9% at 100 °C, suggesting that elevated temperature can strengthen the interaction and further diminish the effective reducing capacity of EGCG. Molecular docking supported this mechanism (Figure 4e,f), showing that the C2 pocket of β–LG provides a well–adapted binding site for EGCG, and the pocket binding score (−7.2) shows a strong binding tendency. The core effect originates from the hydrogen bond network formed between the abundant phenolic hydroxyl groups in EGCG molecules and specific amino acid residues of lactoglobulin. Specifically, the phenolic hydroxyl groups of EGCG form short–distance hydrogen bonds (<3 Å) with TRP19 (2.28 Å) and TYR20 (2.56 Å). In addition, VAL43 (2.33/2.89 Å) and LEU156 (1.99 Å) are positioned near the aromatic rings of EGCG, contributing to hydrophobic interactions and van der Waals contacts that further stabilize the binding complex. These findings demonstrate that the effective antioxidant response of polyphenols in milk–tea systems is significantly lower than in pure tea infusions. Consequently, antioxidant response measured in complex matrices reflects only the detectable antioxidant response in the matrix, which cannot be directly equated with the theoretical content of polyphenol standards. This highlights the necessity of considering matrix interactions when evaluating the measurable antioxidant response of polyphenols in real beverage systems.

### 3.5. Dual–Indicator Recovery Validation for Matrix–Aware Interpretation of Antioxidant Responses

A matrix–matched calibration strategy was established by constructing standard curves for tea infusion addition in milk, together with a dual–indicator evaluation system based on tea infusion recovery and antioxidant response recovery. Green tea infusions with known polyphenol content (814.3 μg/mL) was added to milk at proportions of 0%, 5%, 10%, 20%, 30%, 40%, and 50% (*v*/*v*) to establish the quantitative relationship between tea infusions addition amount and detection response. As shown in Figure 4g,h, both colorimetric (y = 8.34555 + 1.1153x, R^2^ = 0.996) and fluorescent (y = 7.39363 + 1.82694x, R^2^ = 0.989) modes exhibited good linearity over the range of 0–50% tea infusion addition, indicating satisfactory quantitative performance under matrix–matched conditions. More importantly, this calibration step extends the assay from simple antioxidant sensing to matrix–aware evaluation of antioxidant responses, as it better reflects the analytical environment of actual dairy tea systems than EGCG standards measured in simplified solutions.

Based on the above standard curve, spiking recovery experiments were further performed to evaluate method reliability in milk–containing matrices using the dual–indicator system. Tea infusion recovery (%) was calculated as Cdetected/Cadded × 100%, where Cdetected is the tea infusion content obtained from the matrix–matched calibration curve and Cadded is the actual tea infusion addition level. Antioxidant response recovery (%) was calculated as Rmeasured/Rtheoretical × 100%, where Rmeasured is the measured antioxidant response in milk–containing matrices and Rtheoretical is the expected antioxidant response calculated from the corresponding tea infusion at the same addition level. As shown in Table 2, results from both detection modes at four addition levels (5%, 10%, 20%, 40%) showed regular differences. At 10% addition level, tea infusions recovery rates reached 92.97% (colorimetric mode) and 93.80% (fluorescence mode), consistent with the high linearity of standard curves, indicating that the method can support recovery–based estimation of tea infusions addition under matrix–matched conditions. However, the antioxidant response rates at the same level were only 65.08% and 65.66%, approximately 28% lower than tea infusions recovery rates, showing that acceptable recovery of tea infusions addition does not imply full preservation of measurable antioxidant functionality. At low addition levels (5%), the antioxidant response rates further decreased to 44.71–58.85%, suggesting stronger relative masking under protein–excess conditions. These results demonstrate that antioxidant response alone should not be used as the sole basis for interpreting tea–derived input in dairy tea systems. Simultaneous consideration of tea infusion recovery and the antioxidant response provides a more informative analytical basis for evaluating matrix–dependent suppression of antioxidant responses in complex beverage samples.

It should be noted that the tea infusion recovery at the 20% addition level exceeded 130% in both detection modes. This over–recovery does not indicate an enhancement of antioxidant activity, because the corresponding antioxidant response recoveries were still below 100% in both the colorimetric and fluorescence modes. Rather, the over–recovery reflects the uncertainty and possible bias introduced when the signal response is converted into tea infusion content using the matrix–matched calibration curve. In milk–containing systems, protein–polyphenol interactions, matrix heterogeneity, and the non–equivalence between tea infusion content and detectable antioxidant response may all contribute to this deviation. Therefore, the tea infusion recovery should be interpreted as a calibration–derived estimation of added tea infusion, whereas antioxidant response recovery more directly reflects the measurable antioxidant functionality retained in the matrix. The discrepancy between these two indicators further demonstrates the necessity of matrix–aware dual–indicator interpretation in dairy tea systems.

## 4. Conclusions

A rapid dual–mode nanozyme assay based on photoactivated Mn–CNSs was developed for evaluating antioxidant responses in tea beverages. The Mn–CNSs exhibited light–triggered oxidase–like activity toward TMB, enabling synchronized colorimetric and fluorescence readouts within 1 min with LODs of 0.22 and 0.29 μg/mL (EGCG as model antioxidant), good selectivity, and feasibility in tea infusions. Extension to dairy–containing systems revealed matrix–dependent attenuation (56.9–72.2%), attributed to protein–polyphenol interactions supported by molecular docking of β–lactoglobulin–EGCG binding. Matrix–matched calibration with dual–indicator validation demonstrated substantial divergence between tea infusion recovery and antioxidant response recovery in milk matrices, indicating that single–response measurements may underestimate actual polyphenol input. This work highlights a broader methodological imperative to recognize that in complex matrices, analytically detectable signals and functionally accessible contents are not equivalent. By disentangling content from functional detectability, the proposed strategy offers a practical paradigm for transparent, scientifically grounded evaluation of bioactive compounds in real food systems.

## Figures and Tables

**Table 1:** Comparison of oxidase–like nanozyme kinetic parameters using TMB as the substrate.

Material	Vmax (×10^–7^, M s^–1^)	Km (mM)	Reaction Time (min)	Ref.
Fe–N–C SAC	0.06	1.81	15	[32]
Co–SAC	3.45	0.578	10	[33]
N,S–CDs	2.23	0.48	5	[34]
Ag@MnO	1.93	4.79	5	[35]
MnO2@PLNPs	0.158	4.46	10	[36]
Pt/HOFs	0.294	1.54	15	[37]
Fe–NPC	0.5558	0.97	15	[38]
Mn–CNSs	30.51	0.422	1	This work

**Table 2:** Recovery results of tea infusions spiked into milk.

Add Content (%)	Colorimetric			Fluorescence		
	Detected Content (%)	Infusions Recovery Rate (%)	Antioxidant Response Rate (%)	Detected Content (%)	Infusions Recovery Rate (%)	Antioxidant Response Rate (%)
5	4.21	84.08	58.85	3.19	63.88	44.71
10	9.29	92.97	65.08	9.38	93.80	65.66
20	26.49	132.48	92.74	27.13	135.65	94.96
40	43.73	109.24	76.47	46	115.01	80.50

## Data Availability

The original contributions presented in this study are included in the article/Supplementary Material. Further inquiries can be directed to the corresponding authors.

## Supplementary Materials

The following supporting information can be downloaded at: https://www.mdpi.com/article/10.3390/foods15142572/s1. Figure S1 UV–Vis absorption spectra of Mn–CNSs in different solutions; Figure S2 Correlation curve between absorbance and fluorescence intensity of Mn–CNSs in (a) DMSO system and (b) water system; Figure S3 Spectroscopic Characterization of Mn–CNSs under Different Synthesis Conditions; Figure S4 pH responsiveness of Mn–CNSs in (a) colorimetric mode and (b) fluorescence mode; Figure S5 UV–Vis absorption spectra (a) and fluorescence spectra (b) of Mn–CNSs stored in dark vs. light conditions; Figure S6 Fluorescence spectra of Mn–CNs–TMB system; Figure S7 Verification of the fluorescence quenching mechanism; Figure S8 Evaluation of Photoactivated Nanozyme Activity of Mn–CNSs Prepared under Different Synthesis Conditions; Figure S9 Optimization results of Mn–CNSs and TMB dosages; Figure S10 Background absorption evaluation of beverage matrices and validation of antioxidant response determination in tea infusions.

## Abbreviations

The following abbreviations are used in this manuscript:

CNSs	carbon nanosheets
DMF	N,N–dimethylformamide
DMSO	dimethyl sulfoxide
EC	epicatechin (catechin)
EDS	energy–dispersive spectroscopy
EGCG	epigallocatechin gallate
GCG	gallocatechin gallate
HPLC	high–performance liquid chromatography
Km	Michaelis constant
LOD	limit of detection
MES	2–(N–morpholino)ethanesulfonic acid
Mn–CNSs	manganese–doped carbon nanosheets
oxTMB	oxidized 3,3′,5,5′–tetramethylbenzidine
R^2^	correlation coefficient
ROS	reactive oxygen species
TEM	transmission electron microscopy
TMB	3,3′,5,5′–tetramethylbenzidine
UV–Vis	ultraviolet–visible
Vmax	maximum reaction rate
XPS	X–ray photoelectron spectroscopy
XRD	X–ray diffraction
β–LG	β–lactoglobulin
