# Supplementary material for: A Dual–Mode Nanozyme Assay Based on Photoactivated Mn–Doped Carbon Nanosheets for Rapid Evaluation of Antioxidant Responses in Tea Beverages and Dairy Tea Matrices
Source: Foods. 2026 Jul 22;15(14):2572. doi: 10.3390/foods15142572 (PMC13409448; doi:10.3390/foods15142572)
Supplement: Supplementary file 1 [file foods-15-02572-s001.zip › foods-4387737-supplementary.pdf]

Supporting Information

A dual-mode nanozyme assay based on photoactivated Mn-doped carbon nanosheets for rapid evaluation of antioxidant response in tea beverages and dairy tea matrices

Qiongmeng Lu[1], Qiuju He[1], Xiling Fang[1], Zheng Wei[1], Qi Zhang[1], Yaxiong Song[2], Shijie Li [1,2], Shuo Wang[2]**

[1]School of Grain Science and Technology, Jiangsu University of Science and Technology, Zhenjiang, 212100, China.

[2] Tianjin Key Laboratory of Food Science and Health, School of Medicine, Nankai University, Tianjin 300071, China.

**Corresponding Authors:*

E-mail: lsj@just.edu.cn (Shijie Li), wangshuo@nankai.edu.cn (Shuo Wang).

Fax: +86-51184401035

Table of Contents

Figures

Figure S1 UV-Vis absorption spectra of Mn-CNSs in different solutions

Figure S2 Correlation curve between absorbance and fluorescence intensity of Mn-CNSs in (a) DMSO system and (b) water system

Figure S3 Spectroscopic Characterization of Mn-CNSs under Different Synthesis Conditions.

Figure S4 pH responsiveness of Mn-CNSs in (a) colorimetric mode and (b) fluorescence mode

Figure S5 UV-Vis absorption spectra (a) and fluorescence spectra (b) of Mn-CNSs stored in dark vs. light conditions

Figure S6 Fluorescence spectra of Mn-CNs-TMB system

Figure S7 Verification of the fluorescence quenching mechanism

Figure S8 Evaluation of Photoactivated Nanozyme Activity of Mn-CNSs Prepared under Different Synthesis Conditions.

Figure S9 Optimization results of Mn-CNSs and TMB dosages

Figure S10 Background absorption evaluation of beverage matrices and validation of antioxidant response determination in tea infusions

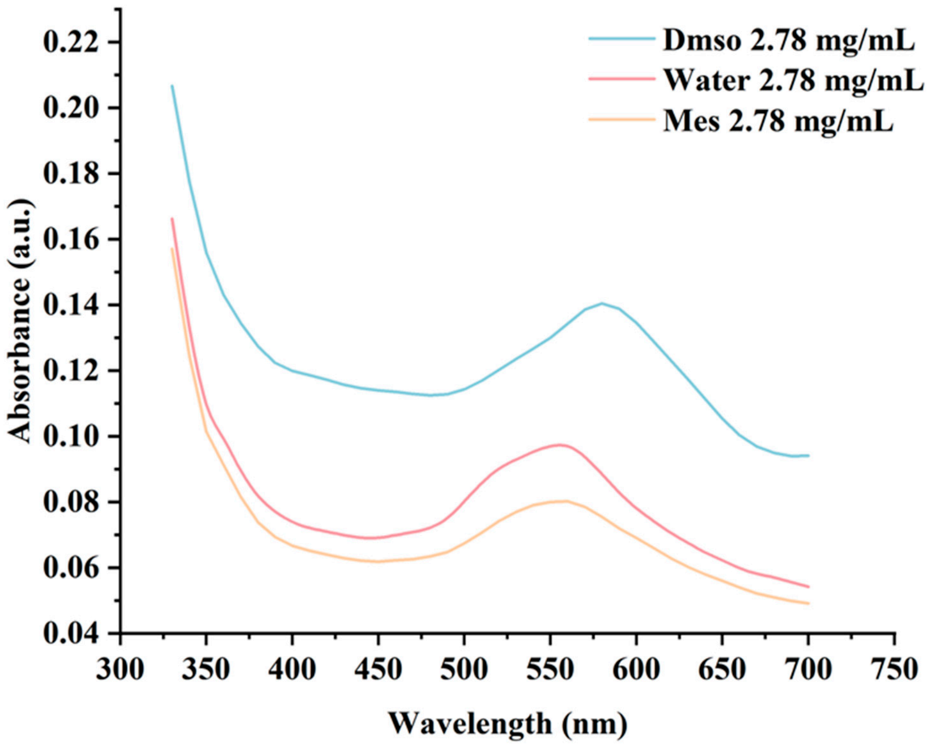

Figure S1 UV-Vis absorption spectra of Mn-CNSs in different solutions

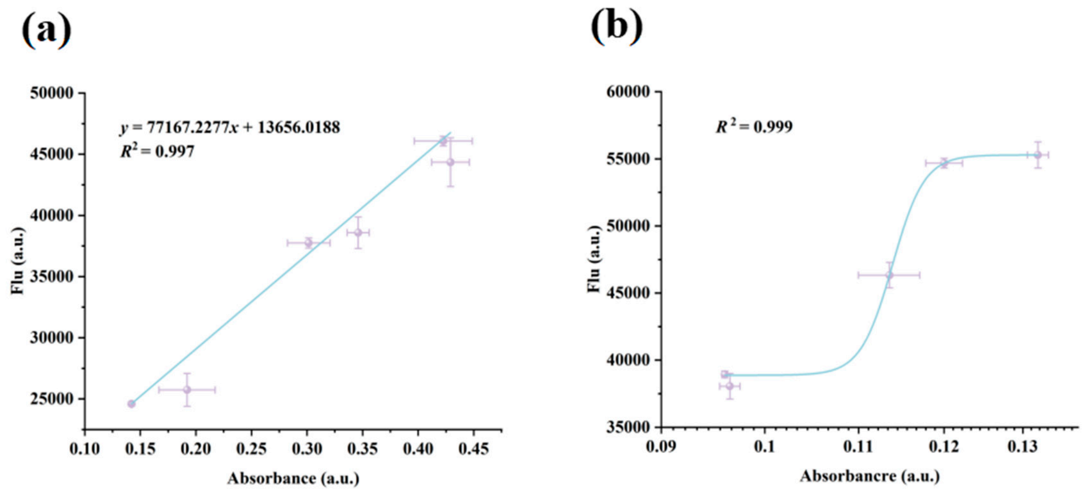

Figure S2 Correlation curve between absorbance and fluorescence intensity of Mn-CNSs in (a) DMSO system and (b) water system

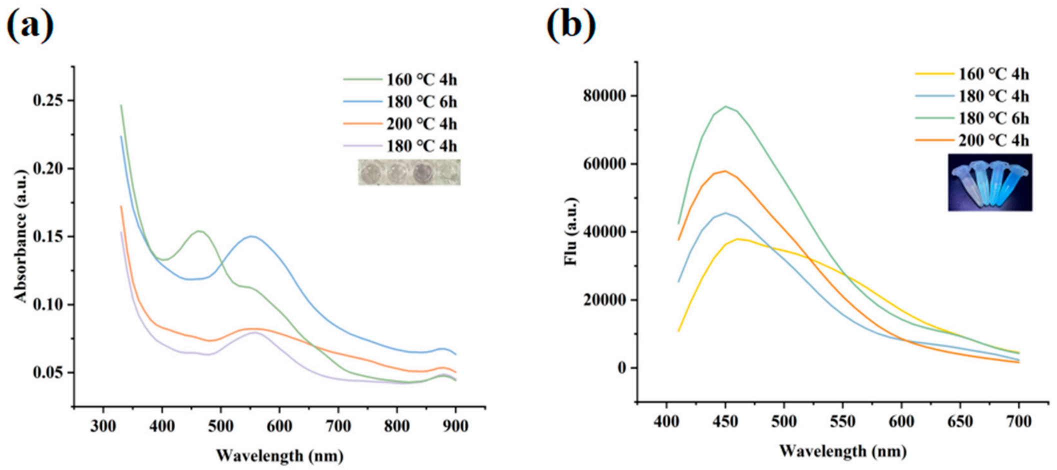

Figure S3 Spectroscopic Characterization of Mn-CNSs under Different Synthesis Conditions. (a) UV–Vis absorption spectra of Mn-CNSs under different synthesis temperature-time conditions; (b) Fluorescence emission spectra of Mn-CNSs under different synthesis temperature-time conditions (inset: the fluorescence photos of the solutions under the corresponding conditions)

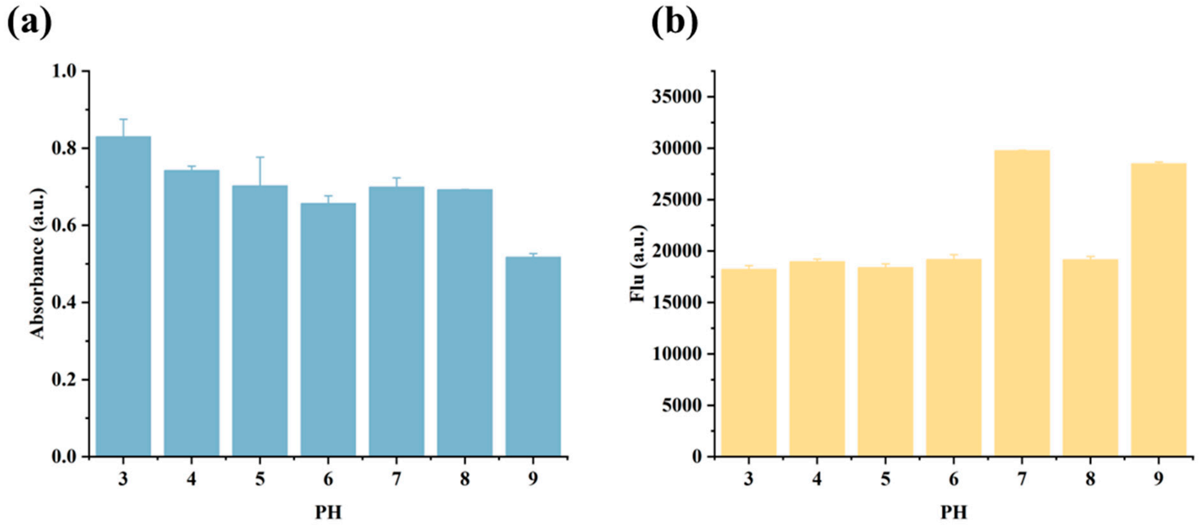

Figure S4 pH responsiveness of Mn-CNSs in (a) colorimetric mode and (b) fluorescence mode

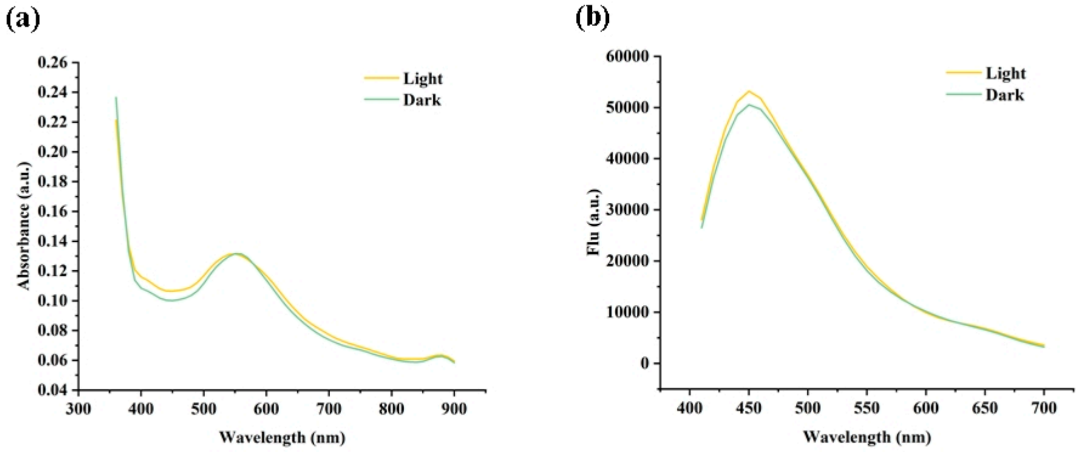

Figure S5 UV-Vis absorption spectra (a) and fluorescence spectra (b) of Mn-CNSs stored in dark vs. light conditions

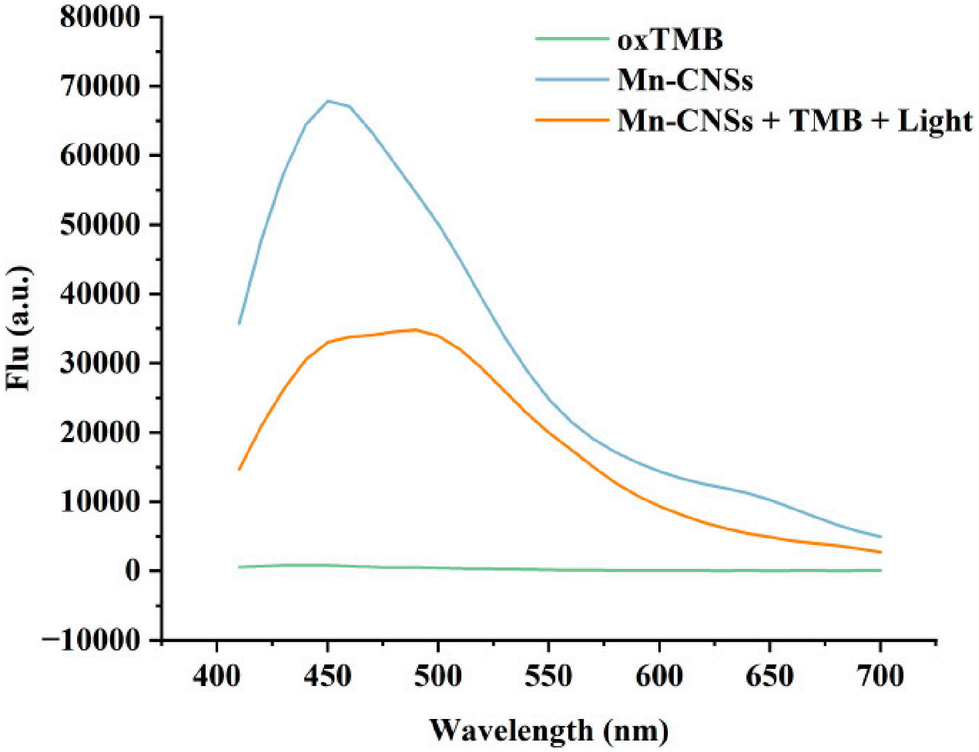

Figure S6 Fluorescence spectra of Mn-CNs-TMB system

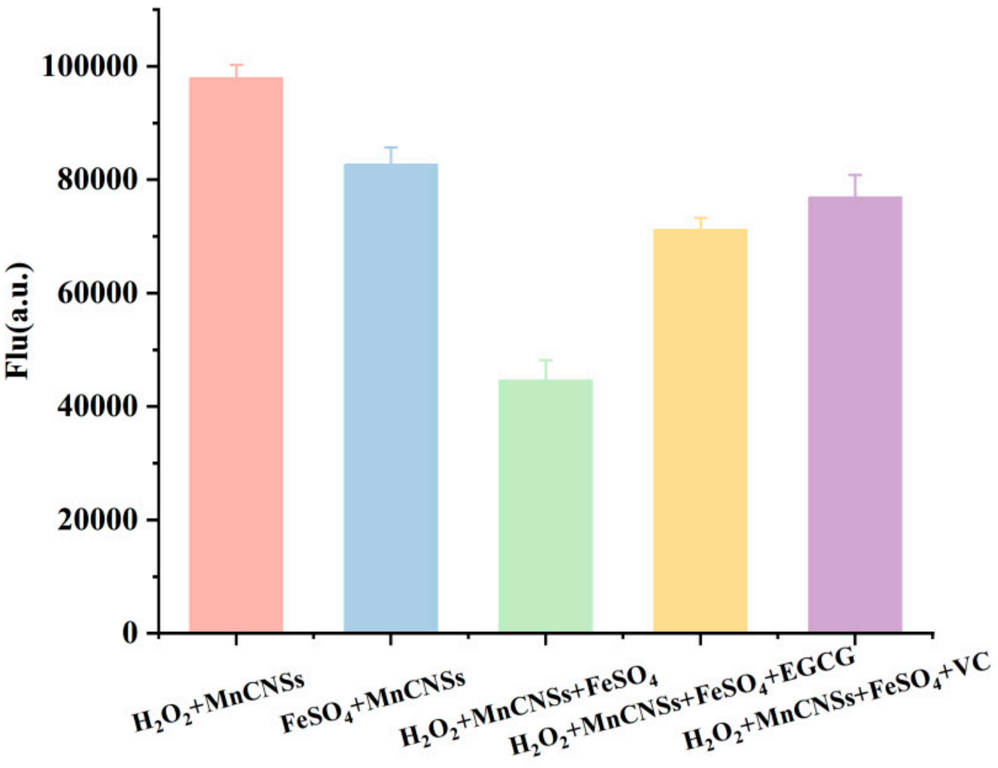

Figure S7 Verification of the fluorescence quenching mechanism

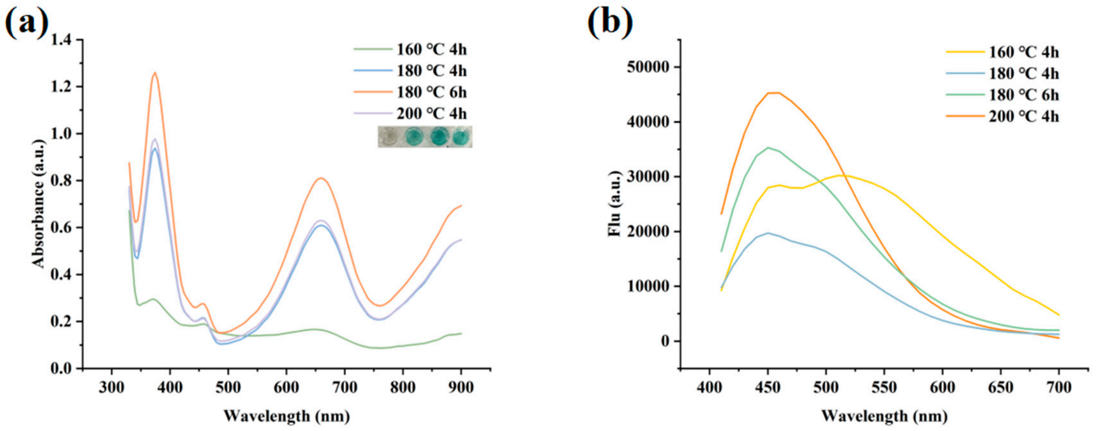

Figure S8 Evaluation of Photoactivated Nanozyme Activity of Mn-CNSs Prepared under Different Synthesis Conditions. (a) UV–Vis absorption and (b) fluorescence emission spectra for the verification of the oxidase activity of Mn-CNSs prepared under different synthesis conditions;

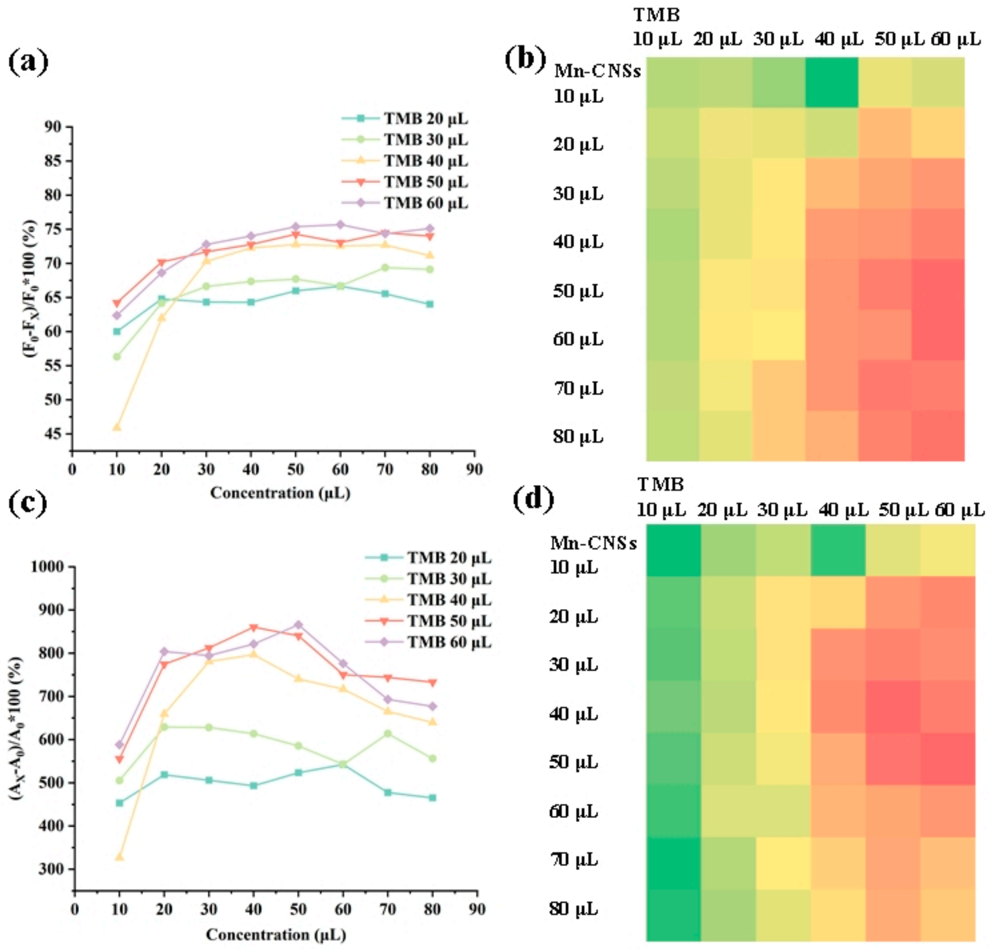

Figure S9 Optimization results of Mn-CNSs and TMB dosages. (a) Fluorescence quenching efficiency under different dosages. (b) Heatmap corresponding to the fluorescence quenching efficiency. (c) Absorbance quenching efficiency under different dosages. (d) Heatmap corresponding to the absorbance quenching efficiency.

The effects of the dosages of Mn-CNSs and substrate TMB on the system performance were further investigated. By systematically adjusting the addition amounts of Mn-CNSs and TMB, the quenching rates of fluorescence and absorbance were analyzed. For fluorescence mode, the fluorescence quenching rate was used as the evaluation index:

$$\frac{F_0\text{-}F_X}{F_0}\times 100\% \qquad \text{(S1)}$$

where F_0 is the fluorescence intensity of the system before light irradiation, and F_x is the fluorescence intensity after light irradiation. The results showed that with the increase of the addition amounts of Mn-CNSs and TMB, the fluorescence quenching rate gradually increased and reached the maximum when Mn-CNSs was 60 μL and TMB was 50 μL. This indicates that the maximum number of reactive oxygen species was generated under this condition, leading to a significant decrease in the system fluorescence intensity.

For colorimetric mode:

$$\frac{A_0\text{-}A_X}{A_0}\times 100\% \qquad \text{(S2)}$$

where A_0 is the absorbance of the system before light irradiation, and A_x is the absorbance after light irradiation. The corresponding heat map was drawn, and the results were consistent with the fluorescence trend. The dosages of Mn-CNSs and TMB have a synergistic effect on the signal intensity, thus determining the optimal reaction ratio as 60 μL of Mn-CNSs and 50 μL of TMB. Under this condition, both the fluorescence and colorimetric signals of the system reach the optimal response, ensuring the high sensitivity and stability of the detection system.

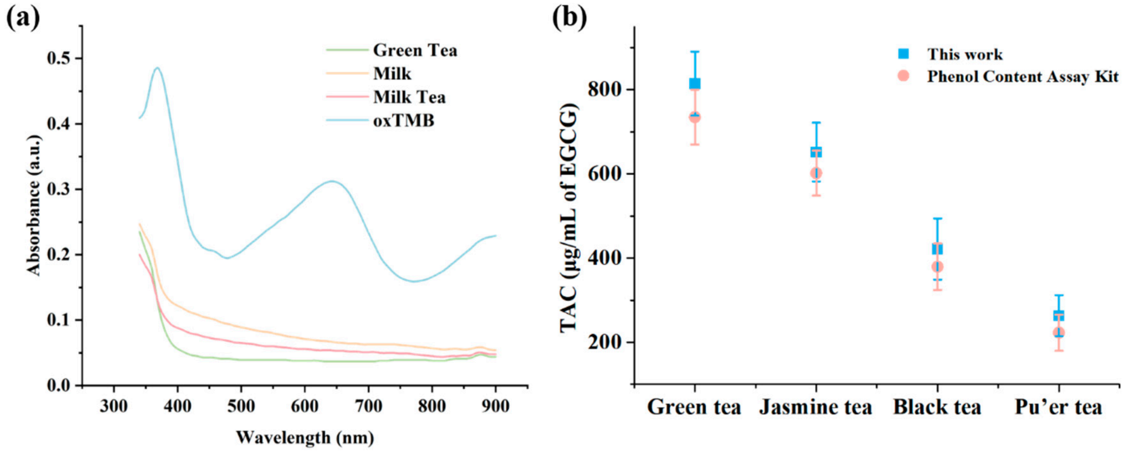

Figure S10 Background absorption evaluation of beverage matrices and validation of antioxidant response determination in tea infusions (a) Background absorption spectra of diluted green tea infusion, milk, and milk tea at 652 nm; (b) Determination of antioxidant response and total phenolic content in different tea infusions.
